# Effects of Erythrodiol on the Antioxidant Response and Proteome of HepG2 Cells

**DOI:** 10.3390/antiox11010073

**Published:** 2021-12-29

**Authors:** Juan Luis Peñas-Fuentes, Eva Siles, Eva E. Rufino-Palomares, Amalia Pérez-Jiménez, Fernando J. Reyes-Zurita, José A. Lupiáñez, Carlos Fuentes-Almagro, Juan Peragón-Sánchez

**Affiliations:** 1 Biochemistry and Molecular Biology Section, Department of Experimental Biology, Campus Las Lagunillas, University of Jaén, 23071 Jaén, Spain; jlpf0004@red.ujaen.es (J.L.P.-F.); esiles@ujaen.es (E.S.); 2 Department of Biochemistry and Molecular Biology I, Faculty of Sciences, University of Granada, Avenida Fuentenueva 1, 18071 Granada, Spain; evaevae@ugr.es (E.E.R.-P.); ferjes@ugr.es (F.J.R.-Z.); jlcara@ugr.es (J.A.L.); 3 Department of Zoology, Faculty of Sciences, University of Granada, Avenida Fuentenueva 1, 18071 Granada, Spain; calaya@ugr.es; 4 Proteomics Unit, Central Service of Support to Research, University of Córdoba (SCAI), 14014 Córdoba, Spain; b72fualc@uco.es

**Keywords:** erythrodiol, HepG2, cytotoxicity, ROS, antioxidant enzymes, GSH, NADPH, proteome

## Abstract

Erythrodiol (EO) is a pentacyclic triterpenic alcohol found in olive tree leaves and olive oil, and it has important effects on the health properties and quality of olive oil. In this study, we characterized the cytotoxic effects of EO on human hepatocarcinoma (HepG2) cells by studying changes in cell viability, reactive oxygen species (ROS) production, antioxidant defense systems, and the proteome. The results reveal that EO markedly decreased HepG2 cell viability without changing ROS levels. The concentrations of glutathione and NADPH were significantly reduced, with selective changes in the activity of several antioxidant enzymes: glutathione peroxidase, glutathione reductase, glucose 6-phosphate dehydrogenase, and 6-phosphogluconate dehydrogenase. Proteomic data reveal that EO led to the complete elimination or decreased abundance of 41 and 3 proteins, respectively, and the abundance of 29 proteins increased. The results of functional enrichment analysis show that important metabolic processes and the nuclear transport of mature mRNA were impaired, whereas AMP biosynthesis and cell cycle G2/M phase transition were induced. The transcription factors and miRNAs involved in this response were also identified. These potent antiproliferative effects make EO a good candidate for the further analysis of its hepatic antitumor effects in in vivo studies.

## 1. Introduction

Pentacyclic triterpenes are a group of plant secondary metabolites with important biological properties for human health and disease prevention. Previous work has studied the compositions of pentacyclic triterpenoids in olive fruits and leaves (*Olea europaea* L.) of different cultivars [1]. Erythrodiol (EO; olean-12-ene-3b,28-diol, C_30_H_50_O_2_; molecular mass, 442.7) is one of the main pentacyclic triterpenic alcohols found in the leaves of the olive tree; it is present at concentrations of 0.6–1.8 mg/g in leaves [1] and 26–90 mg/kg in olive oil [2,3]. EO is synthesized from β-amyrin and is the precursor of oleanolic acid and maslinic acid, which are major pentacyclic triterpenic acids found in the leaves and fruits of the olive tree [1,2,3,4]. These compounds are present in a wide range of plants used in traditional medicine, and EO has been demonstrated to have antioxidant, antiproliferative, and proapoptotic effects on human breast cancer cells [5], HT-29 human adenocarcinoma cells [6], and astrocytoma cells [7]. Moreover, EO has shown anti-inflammatory activity [8,9], vasorelaxant effects [10], and the capacity to modulate cytokine secretion in human cells [11]. Recently, it was reported that uvaol (UO), an isomer of EO, has antiproliferative and proapoptotic effects on HepG2 cells, causing G_0_/G_1_ cell cycle arrest, reactive oxygen species (ROS) production, and AKT/PI3K signaling pathway activation [12].

Many of these effects are initiated by the production of ROS and the regulation of the antioxidant capacity [7,13,14]. To avoid the effects of ROS, cells have different biological defense systems composed of nonenzymatic antioxidants, such as reduced glutathione (GSH) and NADPH, as well as antioxidant enzymes such as superoxide dismutase (SOD), catalase (CAT), glutathione peroxidase (GPX), glutathione S-transferase (GST), glutathione reductase (GSR), glucose 6-phosphate dehydrogenase (G6PDH) and 6-phosphogluconate dehydrogenase (6PGDH). The levels of these antioxidant systems are used as an index of the cellular defense against ROS and oxidative stress.

Monitoring cellular oxidative stress by detecting changes in the levels of ROS is a fundamental aspect of understanding the behavior and development of the carcinogenic process. Oxidative stress is largely associated with the oxidative potential of the mitochondrial membrane. For this reason, the role of changes in the cellular concentration of reducing equivalents in the form of NADPH is vital. In fact, molecules of this reduced coenzyme play fundamental roles in controlling ROS levels and in numerous metabolic processes involved in cell growth and development: they serve as a connection between anabolic and catabolic processes [15,16], are key in maintaining the redox balance [12,17,18], actively participate in the detoxification processes [19], contribute to the maintenance of cellular integrity [18,19] and play an active role in cellular and organic aging [20]. The cellular levels of this reduced coenzyme depend on the enzymatic activity of different NADPH production systems, especially those belonging to the pentose phosphate pathway, G6PDH and 6PGDH, and NADP-dependent isocitrate dehydrogenase (NADP-ICDH). Especially important are changes in its concentration during the vital development of organisms due to its participation in cell growth [21,22,23], cell differentiation [24,25,26], and the maintenance of sensory and nutritional quality [27,28,29].

Recently, the search for potential antitumor compounds derived from natural sources has received much attention. In this regard, several studies have described the effects of pentacyclic triterpenes on different types of human cancer cell lines. The present work aimed to investigate the antiproliferative and antioxidant capacities of EO on HepG2 human hepatocarcinoma cells and determine the changes in the proteome induced by this compound. This cell line is widely used as a cell model for the study of hepatocarcinoma, one of the most common cancers worldwide, and is a good system to study the metabolism of xenobiotics and liver toxicity in vitro [30]. Moreover, it also constitutes a good cell system to investigate the cytoprotective, genotoxic, and antigenotoxic effects of compounds, to understand hepatocarcinogenesis, and to study drug targeting [31,32].

## 2. Materials and Methods

### 2.1. Cell Cultures

The experiments described in this work were approved by the Ethics Committee of the University of Jaén (number: CEIOMGAB-100614-1) and were performed according to the safety and containment requirements for this cell line.

HepG2, a human hepatocellular carcinoma cell line, was provided by “Centro de Investigación Científica” (CIC) of the University of Granada (reference number: ECACC # 85011430, lot CB # 2440). The cells were grown in minimum essential medium (MEM) supplemented with 10% heat-inactivated fetal bovine serum (FBS) and 1% streptomycin/penicillin. They were kept in a CO_2_ incubator at 37 °C, with 100% relative humidity, and 5% CO_2_. Cells were passaged at preconfluent densities using a solution containing 0.05% trypsin and 0.5 mM EDTA. HepG2 cells were seeded in culture dishes at the desired density with the appropriate culture medium.

### 2.2. EO Solution

EO, the compound tested in the experiments, was provided by Sigma (St. Louis, MO, USA), with a purity greater than 95%. A stock solution of EO with a concentration of 20 mg/mL in DMSO was prepared and subsequently diluted in culture medium until reaching the concentrations required for each test.

### 2.3. MTT Assay

The MTT assay was performed as described by Pérez-Jiménez et al. [33]. Briefly, 200 µL samples of cell suspension (1 × 10^4^ cells/well) were cultured in 96-well plates in triplicate. After the adherence of the cells (within 12 h of incubation), EO was added to the wells at a concentration between 0 and 140 µg/mL and maintained for 24, 48, and 72 h. MTT was dissolved in the medium and added to the wells at a final concentration of 0.5 mg/mL. Following 2 h of incubation, the generated formazan was dissolved in DMSO. Absorbance was measured at 570 nm in a multiplate reader (BioTek, Winooski, VT, USA). The percentage of cell viability was calculated using the formula:

% cell viability = (A_0_ − A_T_)/A_0_ · 100 (1)

where A_0_ is the control absorbance (100% cell viability), and A_T_ is the absorbance of cells incubated with different concentrations of EO.

OriginPro8 (OriginLab Corporation, Northampton, MA, USA) was used to perform a dose-response analysis according to the following formula:

Y = A_1_ + (A_2_ − A_1_)/(1 + 10 ^[Log x0-x]p^) (2)

where Log x0 is the center of the curve, *p* is the slope, A_1_ is the lower asymptote, and A_2_ is the upper asymptote in the adjusted model described above.

The concentrations that caused 20%, 50%, and 80% inhibition of cell viability (IC_20_, IC_50_, and IC_80_) were calculated for this analysis. The following tests were carried out using the IC_50_ concentration of EO for an incubation period of 24 h.

### 2.4. Measurement of ROS Production

ROS production was determined using the methods described by Lebel and Bondy [34] adapted to a 12-well plate. This method is based on the use of 2′-7′-dichlorofluorescein diacetate (DCFDA). After DCFDA diffuses into the cell, it is transformed to a non-fluorescent compound that is subsequently oxidized by ROS into 2′-7′-dichlorofluorescein (DCFC), which can be detected by the emission of fluorescence at 535 nm with maximum excitation of 488 nm. Tert-butyl hydroperoxide (TBHP) was used to induce ROS production.

In brief, 12-well plates were seeded at a concentration of 1.5 × 10^5^ cells per well. After one day of growth, EO or DMSO was added to each well. DCFA was added after 24 h of incubation in each experimental condition. In other wells, prior to adding DCFA, TBHP was added and maintained for 1 h at 37 °C. All samples were incubated with DCFA for 30 min at 37 °C, and the fluorescence of each one was measured using a flow cytometer.

### 2.5. Enzyme Activity Assays

#### 2.5.1. Protein Extracts

Samples of liquid culture containing 7.5 × 10^6^ HepG2 cells/flask were seeded in T-75 flasks in triplicate for the two experimental conditions (control and EO). After 24 h of incubation, EO was added to the above-mentioned EO flasks at the IC_50_ concentration and incubated for an additional 24 h. Then, cells were trypsinized, collected, and suspended in EBC buffer (50 mM Tris-HCl pH 8, 120 mM NaCl, 0.5% Nonidet P40) at a concentration of 10 µL/10^6^ cells. Cells were broken down by three cycles of freezing and thawing, and the suspensions were incubated for 1 h on ice. During this time, the samples were stirred in a vortex every 15 min. Afterward, the samples were centrifuged at 15,000× *g* at 4 °C for 15 min. The supernatants were used as protein extracts to determine the protein abundance and enzyme activity.

#### 2.5.2. Superoxide Dismutase (SOD) Assay

SOD (EC 1.15.1.1) was assayed using the spectrophotometric method described by McCord and Fridovich [35] based on the measurement of the cytochrome c reduction rate in the presence of xanthine and xanthine oxidase. The assay medium contained 50 mM potassium phosphate buffer pH 7.8, 0.1 mM EDTA, 50 µL of protein extract, 1.5 mM xanthine, 0.025 mM cytochrome c and 1.25 U/mL xanthine oxidase (XOD). The increase in absorbance per minute at 550 nm was measured for 5 min during incubation at 37 °C. The increases in absorbance were determined in samples with and without protein extract, and the specific activity, expressed as units per milligram of protein, was determined using the two values. One unit of SOD is defined as the amount of enzyme necessary to produce 50% inhibition of the ferricytochrome c reduction rate.

#### 2.5.3. Catalase Assay

Catalase (CAT, EC 1.11.1.6) was assayed using a spectrophotometric method based on the reduction of hydrogen peroxide to water and oxygen according to Aebi [36]. The assay medium contained 50 mM potassium phosphate buffer pH 7.0, 50 µL of protein extract, and 10 mM H_2_O_2_. The increase in absorbance per minute at 240 nm was measured for 2 min during incubation at 37 °C. The specific activity of the enzyme is expressed as units (U) per milligram of protein. One unit of CAT is defined as the amount of enzyme that transforms 1 millimole of H_2_O_2_ per minute under assay conditions.

#### 2.5.4. Glutathione Peroxidase Assay

Glutathione peroxidase (GPX, EC 1.11.1.9) was assayed using the method previously described by Lawrence and Burk [37] adapted to a 96-well plate. The assay medium contained 50 mM potassium phosphate buffer pH 7.0, 0.20 mM NADPH, 1 mM EDTA, 1 mM sodium azide, 1.6 units of glutathione reductase, 1 mM GSH and 10 µL of protein extract. After 5 min of preincubation at room temperature, H_2_O_2_ was added until reaching 1 mM in a cuvette. The increase in absorbance per minute at 340 nm was measured for 5 min at 37 °C. One milliunit (mU) of GPX is defined as the amount of enzyme that transforms 1 nanomole of NADPH per minute under assay conditions.

#### 2.5.5. Glutathione Reductase Assay

Glutathione reductase (GR, EC 1.8.1.7) was assayed using the method previously described by Carlberg and Mannervik [38] adapted to a 96-well plate. The assay medium contained 0.1 M sodium phosphate buffer pH 7.6, 0.1 mM NADPH, 0.5 mM EDTA and 20 µL of protein extract. After 5 min of preincubation at room temperature, GSSG was added to obtain a concentration of 1 mM in the well. The increase in absorbance per minute at 340 nm was measured for 5 min at 37 °C. One milliunit (mU) of GR is defined as the amount of enzyme that transforms 1 nanomole of GSSG per minute under assay conditions.

#### 2.5.6. Glutathione S-Transferase Assay

Glutathione S-transferase (GST, 2.5.1.18) was assayed using the spectrophotometric method of Habig et al. [39] adapted to a 96-well plate, in which the production rate of GS-CNB was determined. The assay medium contained 0.1 M sodium phosphate buffer pH 6.5, 10 µL of protein extract, 1 mM GSH and 1 mM CDNB (1-chloro-2,4-dinitrobenzene). The increase in absorbance per minute at 340 nm was measured for 3 min at 37 °C. One milliunit (mU) of GST is defined as the amount of enzyme that produces 1 nanomole of GS-CNB per minute under assay conditions.

#### 2.5.7. Glucose 6-Phosphate Dehydrogenase and 6-Phosphogluconate Dehydrogenase Enzymes

Glucose 6-phosphate dehydrogenase (G6PDH, EC 1.1.1.49) and 6-phosphogluconate dehydrogenase (6PGDH) were assayed using the spectrophotometric method described by Lupiáñez et al. [24] and Peragón et al. [40] adapted to a 96-well plate, in which the production rate of NADPH was determined.

6PGDH was determined in an assay medium containing 0.05 M Tris-HCl pH 7.4, 20 µL of protein extract, 0.6 mM NADP^+^, 2.5 mM MgCl_2_, and 2 mM 6PG.

G6PDH was determined in an assay medium containing 0.05 M Tris-HCl pH 7.4, 20 µL of protein extract, 0.6 mM NADP^+^, 2.5 mM MgCl_2_, 2 mM 6PG and 2 mM G6P.

The increase in absorbance per minute at 340 nm was measured for 5 min at 37 °C in both types of wells. G6PDH activity was determined by comparing 6PGDH activity to the values obtained in the second type of well. One milliunit (mU) of G6PDH or 6PGDH is defined as the amount of enzyme that transforms 1 nanomole of NADP^+^ per minute under assay conditions.

#### 2.5.8. Determination of Protein Concentration

The protein concentrations in the extracts were determined using the Bradford method [41].

### 2.6. Measurement of Antioxidant Metabolite Concentrations

Control HepG2 cells and HepG2 cells incubated with EO for 24 h were suspended in 0.1% Triton-X and 0.6% sulfosalicylic acid in 0.1 M potassium phosphate buffer and 5 mM EDTA, pH 7.5. For GSSG and GSH determination, 10^6^ cells were suspended in 10 µL of buffer, and for NADPH and NADP^+^ determination, 10^6^ cells were suspended in 30 µL of the buffer. Cells were broken down by two cycles of freezing and thawing. Afterward, the suspensions were incubated for 30 min at room temperature and stirred in a vortex every 10 min. After that, the samples were centrifuged at 30,000× *g* at 4 °C for 4 min. The supernatant was used as protein extract for the determination of metabolites.

#### 2.6.1. Measurement of the Concentrations of GSSG and GSH

We followed the method described by Rahman et al. [42] for the preparation of cell protein extracts and the determination of glutathione concentrations. The assay is based on monitoring the rate of production of 2-nitro-5-thiobenzoic acid (TNB) using 5,5’-dithiobis-2-nitrobenzoic acid (DTNB) as the substrate by monitoring the absorbance at 412 nm. Standard curves for GSH (0–422.4 µM) and GSSG (0–105.6 µM) were previously established to calibrate the rate assay. The contents of total glutathione (GSH + GSSG) and GSSG were determined in 96-well plates. Additionally, the level of reduced GSH was calculated as the difference between total glutathione and GSSG. The glutathione reductase recycling method was used for the determination of GSSG. The cell extracts were treated with 2-vinylpyridine, which covalently reacts with GSH (not with GSSG). The excess 2-vinylpyridine was neutralized with triethanolamine.

#### 2.6.2. Measurement of Concentrations of NADP^+^ and NADPH

The concentrations of NADP^+^ and NADPH were determined using the method described by Zhang et al. [43] adapted to 96-well plates. Protein extracts of control HepG2 cells and HepG2 cells incubated with EO for 24 h were used. For each protein extract, three types of wells were prepared. In the first well, named A1, the NADPH and NADH concentrations were determined by measuring the absorbance of the protein extract at 340 nm and 37 °C. In the second, named A2, NADPH + NADH + NADP^+^ was determined by incubating the assay medium with glucose 6-phosphate dehydrogenase and glucose 6-phosphate. In the third, named A3, glutathione reductase and GSSG were added to consume all NADPH, and the NADH concentration was determined. The absorbance used for NADPH calculation was the absorbance of the A1 well minus that of the A3 well. The absorbance used for NADP^+^ calculation was the absorbance of the A2 well minus that of the A1 well.

### 2.7. nLC–MS Proteomic Method

#### 2.7.1. Protein Extraction

To prepare the samples, cells at 70% confluence were incubated with the IC_50_ concentration of EO for 24 h. They were then washed three times with PBS, scraped off with a cell scraper (Renner), and put into 0.5 mL of lysis buffer containing 8 M urea, 2 M thiourea, 4% CHAPS, 2% IPG buffer, 20 mM dithiothreitol (DTT), 100 mM HCl-Tris and 0.75 mM phenylmethylsulfonyl fluoride (PMSF) (pH 8). These samples were immediately sonicated on ice for 5 min and shaken gently for 1 h at 4 °C. During this time, the samples were moderately shaken in a vortex every 15 min. Lysates were centrifuged at 10,000× *g* for 15 min at 4 °C. The supernatants were used for the nLC–MS proteomic procedure. Three replicates were prepared for each experimental group, each made up of 3 different populations of cells.

The nLC–MS proteomic approach was applied as described by Cuevas-Fernández et al. [44]. Briefly, the samples were concentrated in a unique band by 1D electrophoresis, stained with Coomassie Blue, and digested with trypsin on a polyacrylamide gel to remove possible contaminants. Briefly, protein bands were firstly distained in 200 mM ammonium bicarbonate (AB)/50% acetonitrile (ACN) for 15 min and 5 min in 100% ACN. Protein was reduced by the addition of 20 mM dithiothreitol in 25 mM AB and incubated for 20 min at 55 °C. The mixture was cooled to room temperature, followed by alkylation of free thiols by the addition of 40 mM iodoacetamide in 25 mM AB in the dark for 20 min. After, protein bands were washed twice in 25 mM AB. Proteolytic digestion was performed by addition of trypsin (Promega, Madison, Fitchburg, WI, USA), 12.5 ng/uL of enzyme in 25 mM AB, and incubated at 37 °C temperature overnight. Protein digestion was stopped by the addition of trifluoroacetic acid (TFA) at a 1% final concentration. Digest samples were dried in speedvac. The dried peptides were resuspended in 2% ACN and 0.05% TFA, and 400 ng of each was injected for analysis. nLC–MS was performed on a Dionex Ultimate 3000 RSLCnano system (Thermo Scientific) with a flow of 300 nL/min and an ACN gradient of 3–40% in 0.1% formic acid (FA) for 60 min. The peptides were first trapped in a 5 mm × 300 µm Acclaim Pepmap precolumn and subsequently separated on a 50 cm × 75 µm Acclaim Pepmap nano-column (Thermo Scientific) with a 2 µm particle size. Eluted peptides were analyzed on an Orbitrap Fusion mass spectrometer (Thermo Scientific) equipped with a nanoelectrospray source. A data-dependent acquisition method was applied, first detecting the peptides in the orbitrap detector at a resolution of 120,000, followed by collision-induced dissociation (CID) fragmentation in the ion trap to obtain the MS2 spectra. The mass spectrometer was operated in positive mode. Survey scans of peptide precursors from 400 to 1500 *m*/*z* were performed at 120K resolution (at 200 *m*/*z*) with a 5 × 10^5^ ion count target. Tandem MS was performed by isolation at 1.6 Th with the quadrupole, CID fragmentation with a normalized collision energy of 35, and rapid scan MS analysis in the ion trap. The AGC ion count target was set to 102 and the max injection time was 75 ms. Only those precursors with charge states 2–5 were sampled for MS2. The dynamic exclusion duration was set to 15 s with a 10 ppm tolerance around the selected precursor and its isotopes. Monoisotopic precursor selection was turned on. The instrument was run in top speed mode with 3 s cycles, meaning the instrument would continuously perform MS2 events until the list of non-excluded precursors diminishes to zero or 3 s, whichever is shorter.

#### 2.7.2. Data Analysis

Peptide identification and quantitation were processed using the MaxQuant package, version 1.6.3.4, (Max-Planck-Institute of Biochemistry, Berlin, Germany). Peptide identification was carried out with the Andromeda engine against a database of UniProt *Homo sapiens* (accessed on 25 July 2017, 20168 entries) (www.uniprot.org) with default settings using a MaxLFQ label-free quantification method in MaxQuant software [45,46]. MaxQuant software enables high peptide identification rates, individualized ppb-range mass accuracy, and proteome-wide protein quantification [46]. MS2 spectra were used in the search. Briefly, peptides were generated from tryptic digestion with up to one missed cleavage, with carbamidomethylation of cysteines as a fixed modification and oxidation of methionine as a variable modification. The precursor mass tolerance was 10 ppm, and productions were searched for at 0.6 Da tolerance. Valid peptides were filtered according to the 1% false discovery rate (FDR) q-value. A target-decoy search method was applied, integrating multiple peptide parameters such as length, charge, number of modifications, and identification score into a single quality that acts as statistical evidence of the quality of each spectrum match of peptides. Peptide quantification was performed with the MaxLFQ label-free method. The retention time alignment and identification transfer protocol (“match-between-runs” feature in MaxQuant) was applied. Proteins identified from at least 2 peptides were considered in this analysis.

#### 2.7.3. Label-Free Quantitative Data Analysis in PERSEUS Software

Normalized label-free quantitation (LFQ) intensities obtained with MaxQuant were measured using the Perseus software platform (https://maxquant.net/perseus/, accessed on 1 December 2021) (version 1.6.0.2) [47]. Peak intensities across the whole set of quantitative data for all peptides in the samples were imported from the LFQ intensities of proteins from the MaxQuant analysis and normalized according to the median. Normalized LFQ intensity values were transformed to a base-2 logarithmic scale. Proteins identified in at least 2 replicates were considered. Missing data were imputed by using the lowest value of intensity in a maximum of one replicate per condition (imputed). Protein quantification and calculation of statistical significance were carried out using a two-way Student’s *t*-test, and error correction (adjusted *p*-value) was performed with the Benjamini–Hochberg method. The results obtained were then filtered, and adjusted *p*-value ≤ 0.05 and fold change ≥2 were used as criteria. The mass spectrometry data were deposited in the ProteomeXchange Consortium2 via the PRIDE partner repository [48] with the dataset identifier PXD029865.

#### 2.7.4. Functional Data Analysis

Gene ontology biological enrichment analysis of differentially abundant proteins was conducted with the g:Profiler software (https://biit.cs.ut.ee/gprofiler/gost, accessed on 1 December 2021).

### 2.8. Statistical Analysis

Results are expressed as mean ± standard error of the mean (SEM). The statistical difference between groups was determined through analysis of variance (ANOVA) following the Student’s *t*-test. Differences with *p*-value < 0.05 were considered significant.

## 3. Results

### 3.1. EO Has a Cytotoxic Effect on HepG2 Cells

**Figure 1:**
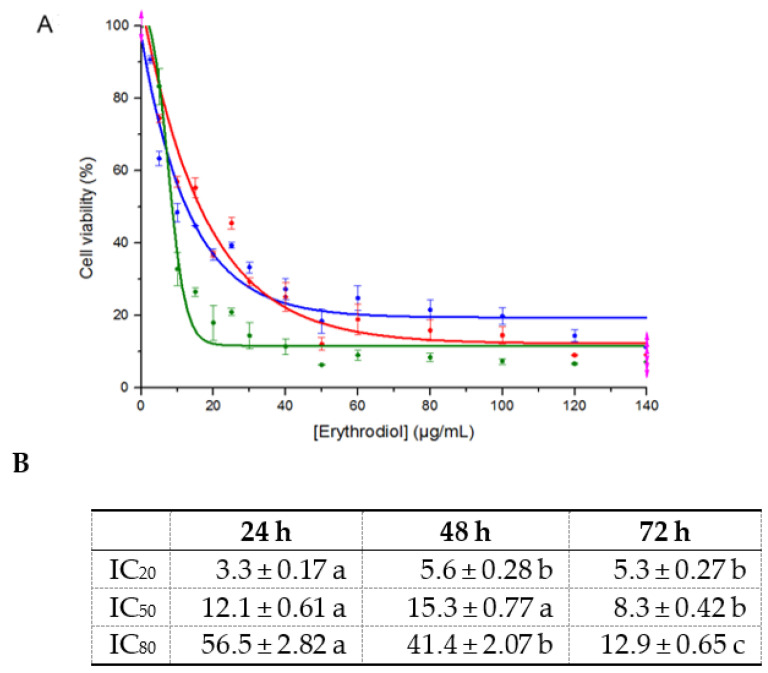
(**A**) Cytotoxicity curve of erythrodiol after HepG2 cells were exposed for 24 (blue), 48 (red), and 72 h (green). (**B**) IC_20_, IC_50_, and IC_80_ values for the HepG2 lines after 24, 48, and 72 h of incubation with erythrodiol. In each row, significant differences (*p* < 0.05) are expressed with different letters, a or b.

The effect of EO on HepG2 cell viability was evaluated by the MTT assay. Figure 1 shows the percentage (%) of cell viability after 24 h of exposure to different concentrations (µg/mL) of EO. This curve is adjusted to a negative sigmoidal curve. The IC_20_, IC_50_, and IC_80_ values were calculated for three incubation periods: 24, 48, and 72 h. Figure 1A shows that EO reduced HepG2 cell viability in a dose-dependent manner. Cell growth was lower than 20% at EO concentrations higher than 30 µg/mL. The concentration at which 50% of cells are viable is the IC_50_ value, which, in these conditions, was 12.1 µg/mL (27.3 µM) after 24 h of EO exposure. The following tests were carried out by incubating cells for 24 h with EO at the above-mentioned IC_50_ concentration.

### 3.2. EO Does Not Change ROS Production in HepG2 Cells

**Figure 2:**
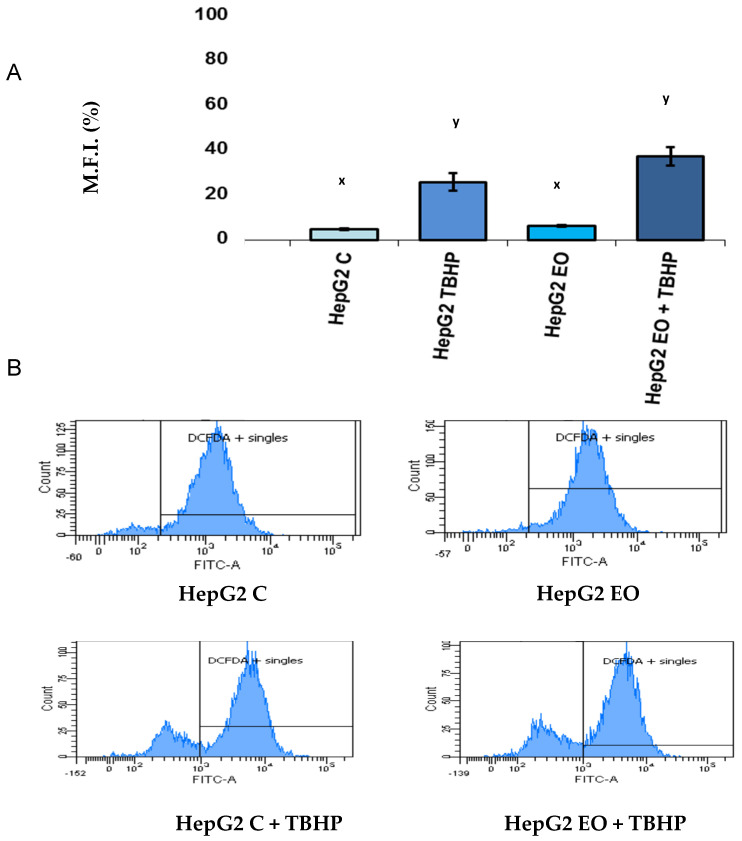
Effect of 24 h incubation with erythrodiol at IC_50_ concentration on the reactive oxygen species (ROS) levels in HepG2 cells. Dichlorofluorescein (DCFC) and tert-butyl hydroperoxide (TBHP) were used. (**A**) Results of the mean fluorescence intensity are expressed in percentage (%). (**B**) The number of DCFC-positive cells and the quantity of emitted fluorescence are shown. Different letters (x or y) indicate different significance levels.

Intracellular ROS levels were measured by flow cytometry after 24 h of exposure to EO. Figure 2 shows the images generated by the flow cytometer during the analysis: the number of DCFC-positive cells and the mean fluorescence intensity are plotted. TBHP was also added to stimulate the production of ROS in control and EO-treated HepG2 cells. The results show that there were no significant differences between the ROS levels in response to EO, both with and without TBHP.

### 3.3. EO Produces Selective Changes in the Specific Activity of Antioxidant Enzymes

Table 1 shows the specific activity of SOD, CAT, GPX, GR, GST, G6PDH, and 6PGDH at saturated substrate concentrations in response to EO treatment in HepG2 cells. SOD, CAT, and GST did not significantly differ after 24 h of incubation with EO. GPX, G6PDH, and 6PGDH significantly increased by 60%, 66%, and 10%, respectively, compared to the control. GR significantly decreased by 26%.

### 3.4. EO Decreases the Levels of Antioxidant Metabolites

Table 2 shows the effects of 24 h of incubation with EO on the levels of GSH, GSSG, NADPH, and NADP^+^ in HepG2 cells. The concentrations of GSH and GSSG underwent significant decreases of 80% and 35%, respectively. In consequence, the total glutathione content ([GSH] + [GSSG]) and glutathione ratio ([GSH]/[GSSG]) decreased by 78% and 70%. The concentrations of NADPH and NADP^+^ decreased by 78% and 30%, respectively. The NADPH/NADP^+^ ratio in EO-treated HepG2 cells was 68% lower than in control cells.

### 3.5. Changes in the Proteome of HepG2 Cells in the Presence of EO

Table 2 cells, we applied a shotgun proteomics approach in which all proteins were hydrolyzed with trypsin, and the resulting peptides were separated by nLC and analyzed by a Thermo Orbitrap Fusion instrument. The proteins corresponding to these peptides were identified and quantified in control HepG2 cells and HepG2 cells incubated with EO for 24 h. The results are shown in Table 3, Table 4 and Table 5. Seventy-three proteins were detected with differential abundance in HepG2 cells treated with EO. Of the 73 differentially abundant proteins, 29 were only found when the cells were incubated with this compound (Table 3), 41 were only detected in the control (Table 4), and 3 had a more than 2-fold lower abundance in HepG2 cells incubated with EO (Table 5). 

**Figure 3:**
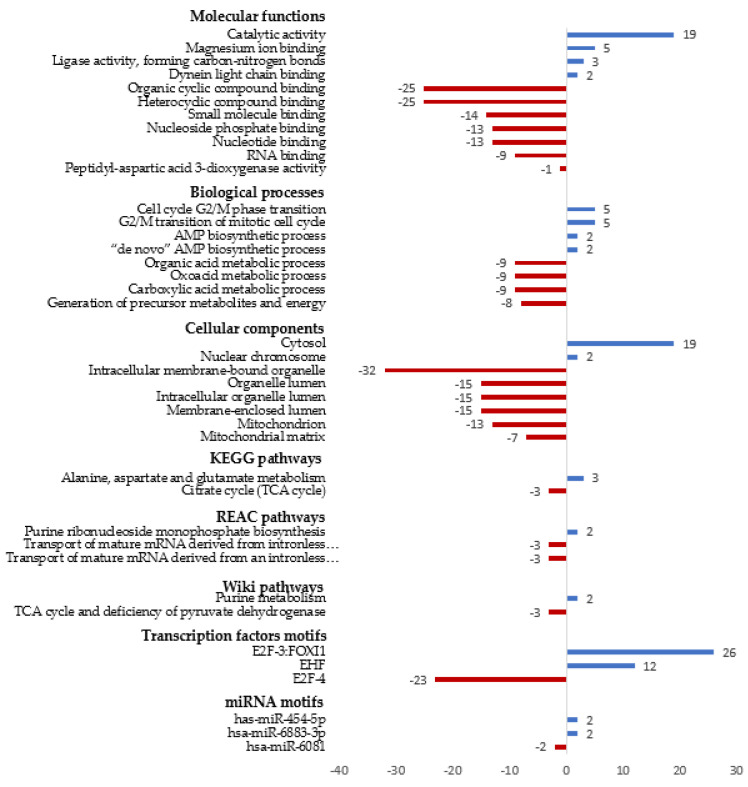
Gene ontology analysis of proteins with differential abundance in HepG2 cells after treatment with EO. In blue proteins only detected after treatment with EO. In red, proteins are only detected in control cells.

For all of the differentially abundant proteins, we performed a gene ontology functional analysis to identify the affected biological processes, molecular functions, cellular components, other biological pathways (Kyoto Encyclopedia of Genes and Genomes (KEGG) pathways, Reactome (REAC) pathways, WiKi pathways) and regulatory motifs in DNA (Transcription Factor (TF) Motifs and microRNA (MIRNA) motifs). We used g:profiler software, which extracts representative functional biological information from large lists of genes and proteins. The results are shown in Figure 3 and Supplementary Tables S1 and S2.

Proteins that were only detected after incubation with EO had the following molecular functions: catalytic activity, ligase activity, and binding to magnesium or dynein light chain. These proteins are involved in the biosynthesis of AMP and the cell cycle G2/M phase transition and are located in the cytosol and nucleus. They are related to the transcription factors E2F-3:FOXI1 and EHF and the miRNA motifs *hsa-miR-6883-3p* and *hsa-miR-454-5p*.

The proteins that were not detected or detected with lower abundance after 24 h incubation with EO are mainly proteins that bind to different organic compounds, such as heterocyclic compounds, RNA, nucleotides, or small molecules. They are involved in several metabolic processes, such as those that generate precursor metabolites and energy and that involve organic acids. They are located in the mitochondria, the lumen, or intracellular organelles or bound to the membrane of organelles. Notably, they are involved in the transport of mature mRNA and their relationship with the transcription factor E2F-4 and *hsa-miR-6081*.

## 4. Discussion

### 4.1. EO Has a Cytotoxic Effect on HepG2 Cells

Cancer cell culture is a good experimental model for screening potential anticancer agents as well as for elucidating the mechanisms of their activities. EO is one of the triterpenoids found in olive leaves and olive oil. Previous studies of this compound have described important activities related to the quality and health effects of olive oil. Specifically, important anticancer activities of EO against human breast cancer cells [5], HT-29 [6], and astrocytoma [7] have been previously reported. In this work, we evaluated the capacity of this compound to inhibit the growth of the HepG2 human hepatocarcinoma cell line. The MTT assay results show that EO has dose-dependent antiproliferative and cytotoxic effects on HepG2 cells and is characterized by an IC_50_ value of 12.1 µg/mL (27.3 µM), which is lower than previously reported values for EO in HT-29 human adenocarcinoma cells (48.8 µM) [6] and UO in HepG2 cells (25.2 µg/mL) [12]. This low value demonstrates that EO has a high capacity for inhibiting the growth of HepG2 hepatocarcinoma tumor cells and, therefore, can act as a hepatic anticancer agent. To our knowledge, this is the first time that the antiproliferative and anticancer effects of EO on hepatic cells have been described, making EO a good candidate for in vivo studies.

### 4.2. EO Does Not Change Intracellular ROS Production, Decreases the Levels of Antioxidant Metabolites, and Produces Selective Changes in the Specific Activity of Antioxidant Enzymes in HepG2 Cells

Based on our results, we can affirm that the effect of EO on HepG2 cell viability is not due to an increase in ROS production. Contrary to other works, we did not find significant increases in the intracellular levels of ROS after 24 h of incubation with EO in either the absence or presence of TBHP, a stimulator of ROS production. This indicates that the mechanism responsible for cytotoxicity in this cell line is different from that induced in cells in which ROS production mediates mitochondrial or kinase-based apoptosis pathways [7,12].

Mammalian cells contain an antioxidant defense system constituted by metabolites and enzymes. GSH and NADPH are the main antioxidant metabolites, and SOD, CAT, GPX, GST, GR, G6PDH, and 6PGDH are the main antioxidant enzymes. In this work, no significant differences in SOD, CAT, or GST activity were observed, although significant depletion of glutathione and NADPH levels was observed. These are the main antioxidant metabolites that support the activities of the antioxidant enzymes. At the same time, the activity of GR significantly decreased, whereas the activities of GPX, G6PDH, and 6PGDH significantly increased. These changes indicate that, although ROS levels and the main antioxidant enzyme activities are maintained in the presence of EO, significant decreases in glutathione and NADPH are produced, leading to a decrease in the antioxidant defense capacity of HepG2 cells. Previous works [49,50] have indicated that the shift in the cellular GSH-to-GSSG redox balance in favor of the oxidized species constitutes an important signal of cell impairment that can lead to the activation of the apoptotic signaling cascade, which may explain the results observed in the antiproliferative activity assay.

### 4.3. Changes in the Proteome of HepG2 Cells in the Presence of EO

We applied a proteomic procedure to identify the proteins and cell functions targeted by EO in HepG2 cells. Similar proteomic approaches have been used in other works to understand the cell response to bioactive compounds [16,50]. Our results show that the abundance of many proteins underwent important changes in response to EO. The majority of differentially abundant proteins disappeared when HepG2 cells were exposed to EO. In particular, these proteins bind to different molecules, such as nucleotides, e.g., GTP (Rhot2, Rab21, Nras, Ranbp2, Rhog); nucleic acids (Wdr33, Mrto4, Rcl1, Taf15, Msh2, Erh, Mrps23, Anapc7, Sars2); proteins (Huwe1, Grpel1, Dnaja3, Nup43) or small molecules. These proteins are related to signal transduction pathways; folding, sorting, and protein degradation; regulation of gene expression; and metabolic processes that generate precursors or energy. This behavior is in agreement with the previously described decrease in glutathione and NADPH concentrations and shows a decrease in all of these pathways and processes and the production of metabolites and energy. A large number of genes that code for these proteins have promoter sequences recognized by the transcription factor E2F-4. E2F is a family of transcription factors that are involved in cell cycle regulation and have several effects on the intrinsic apoptosis pathway [51,52,53]. Moreover, Garneau et al. [54] reported that nuclear expression of E2F-4 induces cell death via multiple pathways in normal human intestinal epithelia. EO can interact with E2F-4, resulting in the repression of its downstream genes.

Moreover, a large number of proteins were also induced. The affected proteins have a catalytic activity or bind to different molecules. The important biological processes that were modulated are the biosynthesis of AMP (Adsl, Adss) and cell cycle G2/M phase transition (Dync1l2, Mastl, Rcc2, Ckap5, Ppp1cb). The transcription factors related to these proteins are E2F-3:FOXI1 and EHF (ETS Homologous Factor). The first one belongs to the E2F family, and EHF is a transcriptional activator that may play a role in regulating epithelial cell differentiation and proliferation [55].

E2F is a family of transcription factors that consist of two classes: one of them, from E2F-1 to E2F-3, drives cell cycle progression by inducing gene expression, and the other, from E2F-4 to E2F-8, impedes cell growth by repressing these genes [56,57]. E2F-3 binds to promoters in a sequence-specific manner and activates the transcription of the corresponding genes. E2F-4 binds to the same sequences but is more important in repression [58]. Both E2F-3 and E2F-4 are presumably involved in the cytotoxic response induced by EO, in which the G2/M phase transition is controlled. Moreover, E2F can promote apoptosis to regulate cell growth. Specifically, it has been established that E2F-3 is required for DNA-damage-induced apoptosis [55]. This can explain the involvement of both transcription factors in the response described in our work: E2F-4 represses HepG2 cell growth, and E2F-3 induces DNA-damage-induced apoptosis.

## 5. Conclusions

The high proliferation capacity of cancerous cells is facilitated by altered metabolic pathways that can support this high rate of proliferation even when the availability of nutrients or oxygen is limited [59]. In conjunction with this, the involvement of metabolic pathways is related to interconnected signaling pathways that are altered in cancer reprogramming. Here, we report that EO alters the concentration of antioxidant metabolites and specific proteins involved in different metabolic and signaling pathways. These effects result in a potent antiproliferative effect on the hepatic cancer cell line. Consequently, EO can be considered a good candidate for analyzing its hepatic antitumor effect in in vivo studies.

## Figures and Tables

**Table 1:** Effects of erythrodiol (EO) on the specific activity at saturated substrate concentrations of superoxide dismutase (SOD), catalase (CAT), glutathione peroxidase (GPX), glutathione reductase (GR), glutathione S-transferase (GST), glucose 6-phosphate dehydrogenase (G6PDH), and 6-phosphogluconate dehydrogenase (6PGDH) in HepG2 cells.

	Control	EO
SOD	2.89 ± 0.05 a	3.09 ± 0.25 a
CAT	54.03 ± 1.91 a	55.82 ± 2.60 a
GPX	1.44 ± 0.17 a	2.31 ± 0.14 b
GR	54.58 ± 6.42 a	40.48 ± 4.67 b
GST	6.78 ± 0.25 a	5.50 ± 0.59 a
G6PDH	16.72 ± 1.74 a	27.79 ± 1.05 b
6PGDH	75.44 ± 1.38 a	82.89 ± 1.52 b

Data are mean ± standard error of the mean and are expressed in milliunits/milligram of protein (SOD, GPX, GR, GST, G6PDH, 6PGDH) or units/milligram of protein (CAT). In each row, comparing control versus EO, values followed by different letters, a or b, are significantly (*p* < 0.05) different.

**Table 2:** Concentrations of GSH, GSSG, NADPH, and NADP^+^ in HepG2 cells incubated with erythrodiol (EO) at IC50 concentration for 24 h.

	Control	EO
[GSH]	2571.00 ± 117.24 a	503.10 ± 38.64 b
[GSSG]	544.4 ± 27.84 a	352.18 ± 14.86 b
[GSH] + [GSSG]	3115.4 ± 150.68 a	855.28 ± 50.91 b
[GSH]/[GSSG]	4.72 ± 0.23 a	1.43 ± 0.08 b
[NADPH]	547.62 ± 21.79 a	121.30 ± 8.14 b
[NADP^+^]	609.26 ± 32.69 a	423.11 ± 24.79 b
[NADPH]/[NADP^+^]	0.90 ± 0.04 a	0.29 ± 0.02 b

Data are mean ± standard error of the mean and are expressed in nmol/g cells. In each row, comparing control versus EO, values followed by different letters, a or b, are significantly (*p* < 0.05) different.

**Table 3:** nLC-MS identified HepG2 proteins that only are found after treatment with erythrodiol.

	Protein IDs ^a^	Gene Name	Protein Name	Score ^b^	Coverage (%) ^c^	Peptides ^d^	kDa	Intensity
1	A0A0G2JY07	Mcm5	DNA replication licensing factor MCM5	9.8013	12.1	7	82.467	90,380,000
2	Q5HZY3	Uchl5	Ubiquitin carboxyl-terminal hydrolase	13.129	22.5	5	37.097	97,660,000
3	A0A0G2K3Q6	Aldoc	Fructose-bisphosphate aldolase C	3.8455	22.5	7	40.484	44,823,000
4	F7EWC1	Vasp	Vasodilator-stimulated phosphoprotein	4.3293	3.5	1	39.485	152,990,000
5	F7FG31	Ctbp1	C-terminal-binding protein 1	6.0241	11.7	4	43.847	67,520,000
6	B4F786	Cd2bp2	CD2 antigen (Cytoplasmic tail) binding protein 2 (Predicted), isoform CRA_a	3.1481	6.2	2	37.54	63,614,000
7	B5DEX9	Arid3a	AT-rich interaction domain 3A	9.361	10.8	5	63.594	79,387,000
8	D3ZAZ0	Eif3m	Eukaryotic translation initiation factor 3 subunit M	102.58	28.6	8	42.516	298,630,000
9	D3ZD89	Naa15	N(alpha)-acetyltransferase 15, NatA auxiliary subunit	22.247	5.2	3	101.01	51,300,000
10	D3ZDK7	Pgp	Glycerol-3-phosphate phosphatase	25.181	17.1	4	34.6	141,480,000
11	D3ZQ74	Plod1	Procollagen-lysine, 2-oxoglutarate 5-dioxygenase 1	197.95	7.1	3	83.612	137,530,000
12	D3ZU74	Dync1i2	Cytoplasmic dynein 1 intermediate chain 2	12.408	4.2	2	68.362	111,420,000
13	D3ZVK3	Trmt6	tRNA (adenine(58)-N(1))-methyltransferase non-catalytic subunit TRM6	4.0587	4	2	55.278	57,655,000
14	D3ZW08	Adsl	Adenylosuccinate lyase	14.433	6.6	3	54.852	56,954,000
15	D4A355	Mastl	Microtubule-associated serine/threonine-protein kinase-like	2.5367	2.3	1	96.233	5,377,500,000
16	D4ADZ9	Pus7	Pseudouridine synthase 7	5.5004	5.8	3	74.64	59,883,000
17	D4AEP0	Adss	Adenylosuccinate synthetase isozyme 2	12.613	17.8	5	50.085	206,830,000
18	F1LPD6	Acaa1b	3-ketoacyl-CoA thiolase A, peroxisomal	5.6309	7.2	3	44.524	123,980,000
19	F1LVV4	Rcc2	Regulator of chromosome condensation 2	29.009	26.9	7	46.652	155,410,000
20	F1LXV3	Stk26	Serine/threonine kinase 26	3.486	5.3	2	46.573	80,258,000
21	F1M949	Ckap5	Cytoskeleton-associated protein 5	5.8607	2.1	3	187.92	53,088,000
22	G3V8R0	RGD1311703	Small acidic protein	28.294	28.2	4	19.961	65,264,000
23	G3V9I9	Srek1	Splicing regulatory glutamine/lysine-rich protein 1	3.0994	5.2	2	69.235	38,385,000
24	P09606	Glul	Glutamine synthetase	50.405	9.9	3	42.267	196,290,000
25	P46413	Gss	Glutathione synthetase	12.553	15.2	5	52.344	170,440,000
26	P62142	Ppp1cb	Ser/thr-protein phosphatase PP1-beta catalytic subunit	5.3181	40.4	10	37.186	266,690,000
27	Q64560	Tpp2	Tripeptidyl-peptidase 2	4.8866	2.5	3	138.29	40,240,000
28	Q6PEC1	Tbca	Tubulin-specific chaperone A	7.2779	17.6	2	12.744	172,870,000
29	Q9ES53	Ufd1l	Ubiquitin fusion degradation protein 1 homolog	6.6969	7.5	2	34.485	105,660,000

^a^ Protein identification number. ^b^ Score corresponding to *p* < 0.05. ^c^ Percentage of peptide sequence homology. ^d^ Number of fragmented peptides with homology.

**Table 4:** nLC-MS identified HepG2 proteins that are found in control cells and not in EO-treated cells.

	Protein IDs ^a^	Gene Names	Protein Names	Score ^b^	Coverage (%) ^c^	Peptides ^d^	kDa	Intensity
1	A0A096MJA9	Asph	Aspartyl/asparaginyl beta-hydroxylase	4.0672	4.3	2	116.29	138,230,000
2	A0A096MK75	Rhog	Rho-related GTP-binding protein RhoG	7.9252	33.1	3	67.165	153,310,000
3	A0A0G2JVW5	Huwe1	HECT-type E3 ubiquitin transferase	16.925	1.2	3	71.614	322,680,000
4	A0A0G2JZA2	Grpel1	GrpE protein homolog 1. mitochondrial	4.6136	16.5	2	21.243	223,840,000
5	A0A0G2K261	Iars2	Isoleucine--tRNA ligase, mitochondrial	35.558	8	5	19.872	129,930,000
6	A0A0G2K4Y1	Dnaja3	DnaJ homolog subfamily A member 3, mitochondrial	5.2058	6.6	2	49.416	63,935,000
7	B0BMT9	Sqrdl	Sulfide:quinone oxidoreductase, mitochondrial	14.818	9.8	3	45.347	93,481,000
8	B0BNB5	Nup43	Nucleoporin Nup43	14.829	8.5	2	46.435	196,970,000
9	B1WBQ7	Msh2	DNA mismatch repair protein Msh2	15.144	5.5	4	112.68	264,890,000
10	B1WC25	Tra2a	Transformer-2 protein homolog alpha	11.134	11	4	58.286	122,620,000
11	B2RYG5	Taf15	TATA-binding protein-associated factor 2N	12.166	14.3	5	55.413	126,240,000
12	B2RYQ5	Erh	Enhancer of rudimentary homolog	14.177	31.7	2	65.673	73,205,000
13	D3ZIN7	Mrps23	28S ribosomal protein S23, mitochondrial	3.558	6.2	2	104.15	151,590,000
14	D3ZIT4	Anapc7	Anaphase-promoting complex subunit 7	14.411	8.5	3	24.857	203,460,000
15	D3ZM09	Sars2	Serine--tRNA ligase, mitochondrial	5.5606	5.4	2	88.596	73,564,000
16	D4A054	Ranbp2	E3 SUMO-protein ligase RanBP2	12.017	3	7	24.163	120,060,000
17	F1LT09	Wdr33	pre-mRNA 3’ end processing protein WDR33	4.5551	2.3	2	43.931	87,020,000
18	F1LTU4	Mrto4	mRNA turnover protein 4 homolog	8.9808	26.4	3	23.397	550,130,000
19	F1M7L9		Uncharacterized protein	7.6448	4.4	4	12.259	63,761,000
20	F1MAA3	LOC100909464	Serine/threonine-protein phosphatase 2A 56 kDa regulatory subunit	6.3291	7.9	3	27.911	76,667,000
21	F7EQ81	Gns	N-acetylglucosamine-6-sulfatase	42.435	5.5	2	74.889	91,214,000
22	G3V7F6	RGD1561590	18 kDa Sin3-associated polypeptide	47.857	27.3	4	15.906	105,570,000
23	G3V7Z1	Rcl1	RNA 3’-terminal phosphate cyclase-like protein	108.33	8.3	2	69.183	64,529,000
24	O70593	Sgta	Small glutamine-rich tetratricopeptide repeat-containing protein alpha	5.0856	12.1	3	69.152	154,570,000
25	P00173	Cyb5a	Cytochrome b5	18.796	25.4	2	341.4	112,790,000
26	P08461	Dlat	Dihydrolipoyllysine-residue acetyltransferase component of pyruvate dehydrogenase complex. mitochondrial	10.385	9.7	4	14.441	341,840,000
27	P12007	Ivd	Isovaleryl-CoA dehydrogenase. mitochondrial	3.6522	4.2	2	20.321	62,345,000
28	P97546	Nptn	Neuroplastin	5.8398	7.9	2	67.603	27,068,000
29	Q04970	Nras	GTPase NRas	10.771	39.2	4	22.162	265,030,000
30	Q06647	Atp5o	ATP synthase subunit O. mitochondrial	12.515	11.7	2	40.81	108,260,000
31	Q3B8N8	Pes1	Pescadillo homolog	15.961	3.2	3	145.41	40,168,000
32	Q5U2N0	Ctps2	CTP synthase 2	3.4722	3.8	2	32.578	93,367,000
33	Q5XI78	Ogdh	2-oxoglutarate dehydrogenase. mitochondrial	8.0893	8	5	62.993	44,703,000
34	Q63396	Sub1	Activated RNA polymerase II transcriptional coactivator p15	11.65	20.5	2	25.811	139,220,000
35	Q63584	Tmed10	Transmembrane emp24 domain-containing protein 10	8.5838	19.2	4	15.355	85,833,000
36	Q6AXT5	Rab21	Ras-related protein Rab-21	60.667	16.6	4	447.65	64,474,000
37	Q6AY02	Rbm17	Splicing factor 45	10.643	14.6	4	50.201	190,840,000
38	Q6AY58	Bcap31	B-cell receptor-associated protein	25.526	8.6	3	41.806	124,930,000
39	Q7TSA0	Rhot2	Mitochondrial Rho GTPase 2	8.3952	7.9	4	60.068	80,485,000
40	Q920L2	Sdha	Succinate dehydrogenase [ubiquinone] flavoprotein subunit. mitochondrial	102.6	7.2	3	19.586	96,290,000
41	Q9WU49	Carhsp1	Calcium-regulated heat-stable protein 1	4.3794	26.5	3	34.157	81,131,000

^a^ Protein identification number. ^b^ Score corresponding to *p* < 0.05. ^c^ Percentage of peptide sequence homology. ^d^ Number of fragmented peptides with homology.

**Table 5:** nLC-MS identified HepG2 proteins that are found in control and EO-treated cells with differential abundances.

	Protein IDs ^a^	Gene Names	Protein Names	Score ^b^	Coverage (%) ^c^	Peptides ^d^	kDa	Intensity (×10^6^)	Fold Change ^e^
1	B0BMT9	Sqrdl	Sqrdl protein	14.818	9.8	3	50.201	190.84	−2
2	Q06647	Atp5o	ATP synthase subunit O. mitochondrial	12.515	11.7	2	23.397	550.13	−2.8
3	Q920L2	Sdha	Succinate dehydrogenase [ubiquinone] flavoprotein subunit. mitochondrial	102.6	7.2	3	71.614	322.68	−2.2

^a^ Protein identification number. ^b^ Score corresponding to *p* < 0.05. ^c^ Percentage of peptide sequence homology. ^d^ Number of fragmented peptides with homology. ^e^ The p values for the fold change were *p* < 0.05.

## Data Availability

The mass spectrometry data were deposited in the ProteomeXchange Consortium2 via the PRIDE partner repository [49] with the dataset identifier PXD029865. Reviewer account details: Username: reviewer_pxd029865@ebi.ac.uk; Password: fAwePjv5.

## Supplementary Materials

The following are available online at https://www.mdpi.com/article/10.3390/antiox11010073/s1, Table S1: Gene ontology analysis of proteins only detected after incubation with EO, Table S2: Gene ontology analysis of proteins not detected or detected at decreased levels after incubation with EO.
