# Supplementary material for: Effects of Erythrodiol on the Antioxidant Response and Proteome of HepG2 Cells
Source: Antioxidants (Basel). 2021 Dec 29;11(1):73. doi: 10.3390/antiox11010073 (PMC8772852; doi:10.3390/antiox11010073)
Supplement: Supplementary file 1 [file antioxidants-11-00073-s001.zip › antioxidants-1513210-supplementary.pdf]

antioxidants MDPI

Supplementary Table S1. Gene ontology analysis of proteins only detected after incubation with EO.

	GO #	*p* value	Genes related
**Molecular functions**			
Catalytic activity	0003824	0.001	MCM5,UCHL5,ALDOC,CTBP1,NAA15,PGP,PLOD1,DYNC1I2,TRMT6,ADSL,MASTL,PUS7,ADSS,STK26,CKAP5,GLUL,GSS,PPP1CB,TPP2
Magnesium ion binding	0000287	0.002	PGP,ADSS,STK26,GLUL,GSS
Ligase activity, forming carbon-nitrogen bonds	0016879	0.011	ADSS,GLUL,GSS
Dynein light chain binding	0045503	0.021	DYNC1I2,GLUL
**Biological processes**			
“de novo” AMP biosynthetic process	0044208	0.004	ADSL,ADSS
AMP biosynthetic process	0006167	0.021	ADSL,ADSS
G2/M transition of mitotic cell cycle	0000086	0.034	DYNC1I2,MASTL,RCC2,CKAP5,PPP1CB
Cell cycle G2/M phase transition	0044839	0.042	DYNC1I2,MASTL,RCC2,CKAP5,PPP1CB
**Cellular components**			
Cytosol	0005829	0.001	MCM5,UCHL5,ALDOC,VASP,CD2BP2,ARID3A,EIF3M,NAA15,DYNC1I2,ADSL,ADSS,RCC2,STK26,CKAP5,GLUL,GSS,PPP1CB,TPP2,TBCA
Nuclear chromosome	0000228	0.033	MCM5,UCHL5
**KEGG pathways**			
Alanine, aspartate and glutamate metabolism	00250	0.007	ADSL,ADSS,GLUL
**REAC pathways**			

Purine ribonucleoside monophosphate biosynthesis	R-HSA-73817	0.045	ADSL,ADSS
**Wiki pathways**			
Purine metabolism	WP4792	0.037	ADSL,ADSS
**Transcription factors motifs**			
E2F-3:FOXI1	M08208	0.0149	MCM5,UCHL5,ALDOC,VASP,CTBP1,CD2BP2,ARID3A,EIF3M,NAA15,PGP,PLOD1,DYNC1I2,TRMT6,ADSL,MASTL,PUS7,ADSS,RCC2,STK26,CKAP5,SREK1,GLUL,GSS,PPP1CB,TPP2,TBCA
EHF	M02052_1	0.022	MCM5,UCHL5,VASP,CTBP1,CD2BP2,EIF3M,PGP,DYNC1I2,TRMT6,ADSL,MASTL,ADSS
**miRNA motifs**			
hsa-miR-6883-3p	hsa-miR-6883-3p	0.019	UCHL5,ALDOC
has-miR-454-5p	hsa-miR-454-5p	0.020	UCHL5,ALDOC

Supplementary Table S2. Gene ontology analysis of proteins not detected or detected at decreased levels after incubation with EO.

	GO #	*p* value	Genes related
**Molecular functions**			
Heterocyclic compound binding	1901363	0.002	RHOG,GRPEL1,IARS2,DNAJA3,MSH2,TRA2A,TAF15,ERH,MRPS23,SARS2,RANBP2,WDR33,MRTO4,CYB5A,IVD,NRAS,PES1,CTPS2,OGDH,SUB1,RAB21,RBM17,RHOT2,SDHA,CARHSP1
Organic cyclic compound binding	0097159	0.0023	RHOG,GRPEL1,IARS2,DNAJA3,MSH2,TRA2A,TAF15,ERH,MRPS23,SARS2,RANBP2,WDR33,MRTO4,CYB5A,IVD,NRAS,PES1,CTPS2,OGDH,SUB1,RAB21,RBM17,RHOT2,SDHA,CARHSP1
RNA binding	0003723	0.0034	IARS2,TRA2A,TAF15,ERH,MRPS23,SARS2,RANBP2,WDR33,MRTO4
Peptidyl-aspartic acid 3-dioxygenase activity	0062101	0.0219	ASPH
Nucleotide binding	0000166	0.0249	RHOG,GRPEL1,IARS2,DNAJA3,MSH2,ERH,SARS2,IVD,NRAS,CTPS2,RAB21,RHOT2,SDHA
Nucleoside phosphate binding	1901265	0.0251	RHOG,GRPEL1,IARS2,DNAJA3,MSH2,ERH,SARS2,IVD,NRAS,CTPS2,RAB21,RHOT2,SDHA
Small molecule binding	0036094	0.0251	RHOG,GRPEL1,IARS2,DNAJA3,MSH2,ERH,SARS2,IVD,NRAS,CTPS2,OGDH,RAB21,RHOT2,SDHA
**Biological processes**			
Generation of precursor metabolites and energy	0006091	0.012	ASPH,NUP43,MSH2,RANBP2,CYB5A,DLAT,OGDH,SDHA
Carboxylic acid metabolic process	0019752	0.023	IARS2,NUP43,SARS2,RANBP2,CYB5A,DLAT,IVD,CTPS2,OGDH
Oxoacid metabolic process	0043436	0.029	IARS2,NUP43,SARS2,RANBP2,CYB5A,DLAT,IVD,CTPS2,OGDH
Organic acid metabolic process	0006082	0.037	IARS2,NUP43,SARS2,RANBP2,CYB5A,DLAT,IVD,CTPS2,OGDH
**Cellular components**			
Mitochondrion	0005739	0.0007	GRPEL1,IARS2,DNAJA3,MRPS23,SARS2,RANBP2,CYB5A,DLAT,IVD,OGDH,BCAP31,RHOT2,SDHA
Membrane-enclosed lumen	0031974	0.0007	ASPH,GRPEL1,IARS2,DNAJA3,NUP43,MSH2,TRA2A,TAF15,ANAPC7,SARS2,RANBP2,WDR33,MRTO4,GNS,RCL1

Intracellular organelle lumen	0070013	0.0008	ASPH,GRPEL1,IARS2,DNAJA3,NUP43,MSH2,TRA2A,TAF15,ANAPC7,SARS2,RANBP2,WDR33,MRTO4,GNS,RCL1
Organelle lumen	0043233	0.0008	ASPH,GRPEL1,IARS2,DNAJA3,NUP43,MSH2,TRA2A,TAF15,ANAPC7,SARS2,RANBP2,WDR33,MRTO4,GNS,RCL1
Mitochondrial matrix	0005759	0.0009	GRPEL1,IARS2,DNAJA3,SARS2,DLAT,IVD,OGDH
Intracellular membrane-bound organelle	0043231	0.009	ASPH,RHOG,GRPEL1,IARS2,DNAJA3,NUP43,MSH2,TRA2A,TAF15,ERH,MRPS23,ANAPC7,SARS2,RANBP2,WDR33,MRTO4,GNS,RCL1,SGTA,CYB5A,DLAT,IVD,NRAS,PES1,OGDH,SUB1,TMED10,RAB21,RBM17,BCAP31,RHOT2,SDHA
**KEGG pathways**			
Citrate cycle (TCA cycle)	00020	0.0167	DLAT,OGDH,SDHA
**REAC pathways**			
Transport of mature mRNA derived from an intronless transcript	R-HSA-159231	0.006	NUP43,RANBP2,WDR33
Transport of mature mRNA derived from intronless transcripts	R-HSA-159234	0.0067	NUP43,RANBP2,WDR33
**Wiki pathways**			
TCA cycle and deficiency of pyruvate dehydrogenase	WP2453	0.0042	DLAT,OGDH,SDHA
**Transcription factors motifs**			
E2F-4	M07084	0.002	ASPH,RHOG,GRPEL1,IARS2,DNAJA3,NUP43,MSH2,TRA2A,TAF15,ERH,MRPS23,ANAPC7,SARS2,RANBP2,WDR33,MRTO4,GNS,RCL1,SGTA,CYB5A,DLAT,IVD,NPTN
**miRNA motifs**			
hsa-miR-6081	hsa-miR-6081	0.032	ASPH,RHOG
